# A Study about Kalman Filters Applied to Embedded Sensors

**DOI:** 10.3390/s17122810

**Published:** 2017-12-05

**Authors:** Aurélien Valade, Pascal Acco, Pierre Grabolosa, Jean-Yves Fourniols

**Affiliations:** 1 LAAS-CNRS, Université de Toulouse, CNRS, INSA, 31031 Toulouse, France; pacco@laas.fr (P.A.); fourniols@laas.fr (J.-Y.F.); 2 Institut Méditerranéen d’Enseignement et de Recherche en Informatique et Robotique, 66004 Perpignan, France; pierre.grabolosa@imerir.com

**Keywords:** smart sensors, Kalman filters, algorithm complexity, IMU, compensation

## Abstract

Over the last decade, smart sensors have grown in complexity and can now handle multiple measurement sources. This work establishes a methodology to achieve better estimates of physical values by processing raw measurements within a sensor using multi-physical models and Kalman filters for data fusion. A driving constraint being production cost and power consumption, this methodology focuses on algorithmic complexity while meeting real-time constraints and improving both precision and reliability despite low power processors limitations. Consequently, processing time available for other tasks is maximized. The known problem of estimating a 2D orientation using an inertial measurement unit with automatic gyroscope bias compensation will be used to illustrate the proposed methodology applied to a low power STM32L053 microcontroller. This application shows promising results with a processing time of 1.18 ms at 32 MHz with a 3.8% CPU usage due to the computation at a 26 Hz measurement and estimation rate.

## 1. Introduction

Since the “Smart Dust” project [1] from Berkeley in 1999, Smart Sensors technologies and the Internet of Things (IoT) have been growing fields of research, focussing on data collection and interpretation [2]. The currently most used paradigm is to measure raw data from the sensor and send the data to the cloud, or a computer, in order to be processed by complex algorithms [3,4]. Another school behind those devices is to process sensor data within the sensor and provide the user with a readily usable, filtered and normalized measurement. The resulting lower data throughput and lower latency allow for lower consumption in wireless applications and easier measurement usage. The ultimate goal would be compensating all sensor dispersion and deviation, independently of environmental conditions, resulting in smart self correcting sensors.

The current and developing processing capabilities of sensors and the growing demand for Smart Sensors, in a wide array of fields from hobbies to industrial automation, call for new embedded, in-line, real-time data processing applications. One of these applications is automatic and continuous sensor calibration [5] with correction over time. Such a sensor does accelerate the manufacturing processes and provides more precise measurements over time, without costly human intervention or sensor replacement due to age related deviation.

State of the art lab sensors, such as pH-meters or spectrometers, use systematic manual recalibration procedures before each measurement to ensure good environmental conditions, dispersion, and deviation compensation. This method is, by definition, not applicable to embedded sensors providing continuous and autonomous measurement without any external intervention.

As most sensing elements are sensitive to multiple physical parameters, it is theoretically possible to automatically enhance measurement precision by merging complementary sensors data using multi-physical parameters estimation. One such common method was initially presented by Kalman in 1960 [6] and is known as the Kalman filter. This filter is a specialization of Bayesian filters [7] restricted to discrete time, linear systems with Gaussian noise, and a state space model of the system. Such a filter allows estimating the internal state of a system based on its measurements and model.

For the last 50 years, Kalman filters and its extensions for non-linear systems, Extended Kalman Filter (EKF) and Unscented Kalman Filter (UKF) [8], have been wildly used in various applications such as satellites, spacecrafts or planes to help automatic control of the systems. Due to its computing complexity, it has however not been wildly used in embedded systems until recent improvements to microcontrollers technologies and processing power. Such applications includes smartphones or drones for pose estimation.

This paper discusses a multi-sensor and multi-physical model coupled with a Kalman filter to achieve precise continuous estimation of a physical value without environmental bias while constrained to low processing power of embedded systems. Additionally, an automatic system re-calibration procedure in known conditions is derived.

The remainder of this paper is organized as follows: Section 2 summarizes the technical background used for this work; Section 3 exposes the proposed methodology; Section 4 applies the methodology to a 2D orientation estimation problem based on inertial measurement units; Section 5 exposes and discusses the results obtained with the application; Section 6 references the used materials and methods; and Section 7 describes the conclusions and the future of this work.

## 2. Technical Background

A multi-physical system model is a multiple input/multiple outputs (MIMO) system with physical values for some of its inputs, calibration values for some of its parameters and multi-parametric equations relying on these values for its measurement outputs. Such a system can be described using the state space representation using two equations: an evolution equation and an output/measurement equation.

### 2.1. Modeling the System

The state space representation [9] is a common MIMO system modeling toolkit. It relies on three vectors and two equations to describe the relation between the system commands, its state, and its outputs:

- an input vector U, containing all the system known inputs;
- a state vector X, containing the system internal state, which will evolve depending on the inputs;
- an output vector Y, containing the system outputs/measurement;
- an evolution Equation (1), describing the evolution of the internal state of the system, depending on the previous state and the command input vector; and
- a measurement Equation (2), describing the measurements at the output of the system depending on its state and its command input.
  (1) $$X_{k+1}=f\left(X_{k},U_{k}\right)$$
  (2) $$Y_{k}=h\left(X_{k},U_{k}\right)$$

Linear Equations (1) and (2) can be written as matrix operations, as illustrated in Equations (3) and (4).

(3) $$X_{k+1}=\left[A\right]\cdot X_{k}+\left[B\right]\cdot U_{k}$$

(4) $$Y_{k}=\left[C\right]\cdot X_{k}+\left[D\right]\cdot U_{k}$$

From this point, the system model can be classified into three categories:

- linear systems, using linear evolution (Equation (3)) and linear measurement functions (Equation (4));
- non-linear systems, using non-linear evolution (Equation (1)) and measurement functions (Equation (2)); and
- mixed systems using, for instance, a linear evolution function (Equation (3)) and a non-linear measurement function (Equation (2)) (or Equations (1) and (4)).

This representation can be applied to large systems composed of multiple subsystems, whether those are coupled, uncoupled, or unidirectionally coupled-respectively dependent, independent, or semi-dependent. Unless coupled, the larger system can be decomposed into the sum of its uncoupled subsystems, enabling major optimizations for computational complexity [10].

### 2.2. Kalman Filters

As exposed earlier, Kalman filters rely on the state representation of a system. They are specialized Bayesian estimators for linear systems with discrete time and Gaussian noises. To do the estimation, the Kalman filter updates Equations (3) and (4) to Equations (5) and (6), where $V_{k}$ and $W_{k}$ are state and measurement noise vectors.

(5) $$X_{k+1}=\left[A\right]\cdot X_{k}+\left[B\right]\cdot U_{k}+V_{k}$$

(6) $$Y_{k}=\left[C\right]\cdot X_{k}+\left[D\right]\cdot U_{k}+W_{k}$$

**Figure 1:**
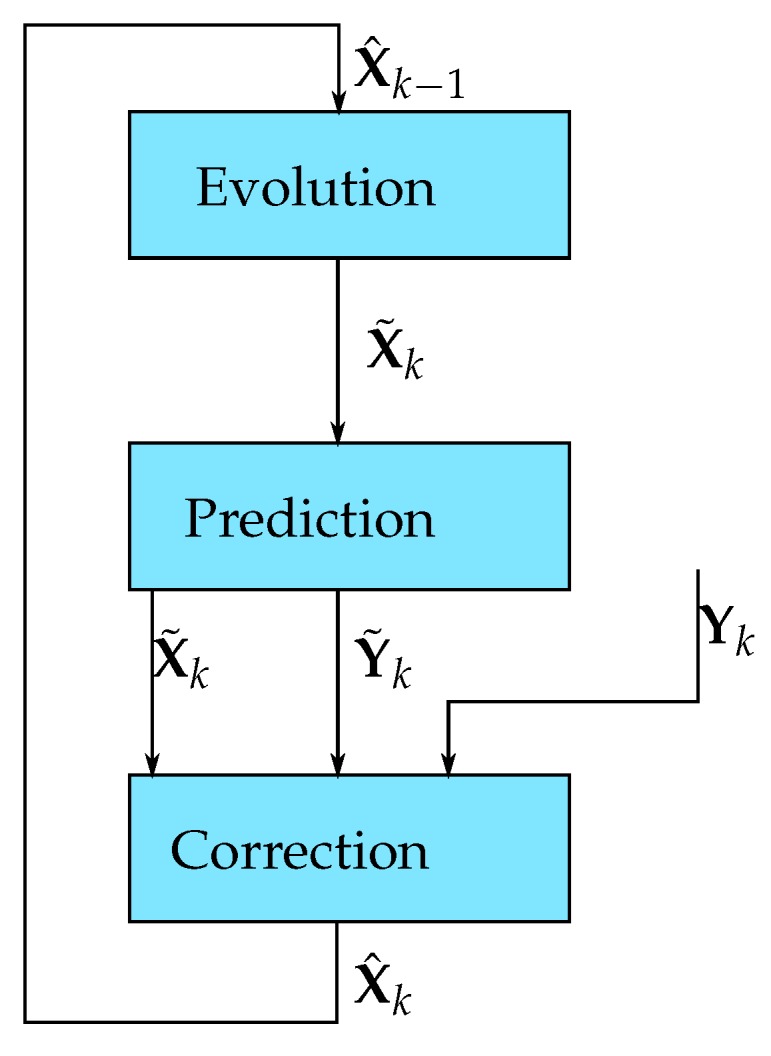
The Kalman filter recursive process.

The Kalman filter is a recursive filter (i.e., it uses the output of its previous corrected estimation to process the next one). This process can be represented by Figure 1, with each step detailed by Table 1.

This filter tends to reduce the quadratic error of ${\hat{X}}_{k}$. Once the model is fixed, the fitting of the filter is done by adjusting the values of covariance matrices of $V_{k}$ and $W_{k}$, [Q] and [R] respectively, and the initial estimated covariance of X, $\left[P_{0}\right]$.

This filter has excellent estimation performances on well known linear system. For non-linear system, extensions have been developed, the best known being the Extended Kalman Filter (EKF) and the Unscented Kalman Filter (UKF).

#### 2.2.1. The Extended Kalman Filter

**Figure 2:**
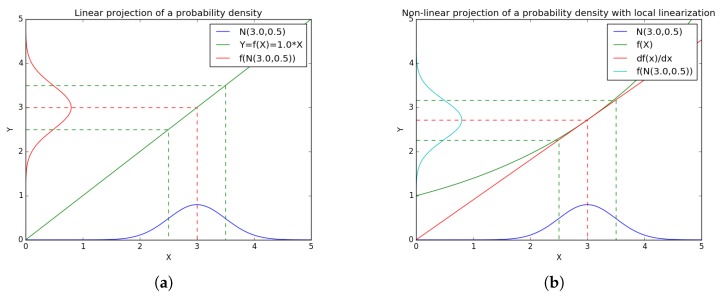
Linear projection methods for: (**a**) linear system (Kalman projection); and (**b**) non-linear system local linearization (EKF projection).

The first, and easiest to understand, method to handle a non-linear system is the well-known EKF. To estimate a non-linear system, the EKF filter does a local linearization of the system equations around the current estimated state. The probability densities of the estimated state and measurement vectors are obtained through the usage of the non-linear function for the mean value and the multiplication of the standard deviation of the estimated state by the corresponding linear function (Figure 2). As the Kalman filter, the EKF considers the system as noisy, the non-linear equations for the system (Equations (1) and (2)) are transformed into Equations (7) and (8).

(7) $$X_{k+1}=f\left(X_{k},U_{k},V_{k}\right)$$

(8) $$Y_{k}=h\left(X_{k},U_{k},W_{k}\right)$$

In the case of state spaces represented systems, this is done by the computation of the Jacobian matrices of the evolution function (Equation (7)). $\left[F_{X}\right]$ is the state relative Jacobian and $\left[F_{V}\right]$ is the noise relative Jacobian.

Using the same pattern for the measurements, the state relative Jacobian will be named $\left[H_{X}\right]$ and the noise relative one $\left[H_{W}\right]$.

The whole estimation process is expose in Table 2.

This method’s main asset is its simplicity, as the operations are identical to the standard Kalman filter. The only addition is the computation of Jacobian matrices and the usage of non-linear functions for evolution and measurement predictions.

A known limitation is the slower convergence and instability of EKF compared to UKF when applied to systems with high non-linearities [11].

#### 2.2.2. The Unscented Kalman Filter

To avoid large non-linearity estimation problems due to the local linearization used by the EKF, the UKF [12] was developed based on a mix between the Particle filter [13] and the Kalman filter. The method is based on the Unscented Transform to propagate the probability density directly through the non-linear function.

##### The Unscented Transform

The Unscented Transform (Appendix A) replaces the approximated linear projection of the Gaussian noise through the linear function by the projection of 2n+1 weighted points, *n* being the size of the state vector, through the non-linear function. The mean and standard deviation of the weighted projected points is then computed to approximate the new, more precise, Gaussian density function at the output [8,14,15].

**Figure 3:**
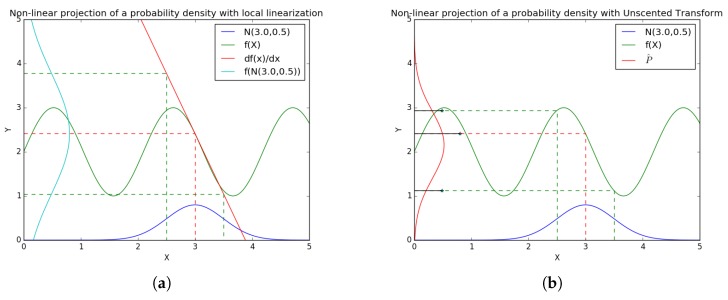
Non-Linear projection methods for: (**a**) local linearization system (EKF projection); and (**b**) non-linear weighted projections (Unscented Transform projection).

As shown in Figure 3, the estimated probability density used by the UKF is far more precise than the EKF in the case of highly non-linear systems: the output of the studied function is limited to [1;2], the corresponding probability density should be zero outside these boundaries. In the case of the local-linearization method with the selected input probability density, the probability of a value higher than 2 is still important. On the other hand, with the Unscented Transform projection, the estimated probability density is far more coherent with the theoretical one.

Using this method, the computation for the UKF is decomposed into three steps: system evolution, measurements projection and correction.

##### System Evolution

- Generate a weighted point set for the following state estimation.
  (9) $$X_{k}=\left[{\hat{X}}_{k},{\hat{X}}_{k}+\sqrt{\left(n+\lambda \right)\left[{\hat{P}}_{k}\right]},{\hat{X}}_{k}-\sqrt{\left(n+\lambda \right)\left[{\hat{P}}_{k}\right]}\right]$$
  With $X_{k}$ a [n,2n+1] matrix representing the 2n+1 states to propagate, weighted with $\omega^{c}$ and $\omega^{\mu }$ previously computed according to Appendix A. The spread of those states around the mean value is adjusted using the λ parameter. The square root of a matrix is defines as $\left[B\right]=\sqrt{\left[A\right]}\;\Leftrightarrow \;\left[B\right]\cdot \left[B\right]=\left[A\right]$, as explained in [16], and implies [A] is a square matrix.
- Propagate the state through the evolution function
  (10) $${\tilde{X}}_{k+1}^{\left(i\right)}=f\left(X_{k}^{\left(i\right)},U_{k},0\right),i\in \left[0,2n\right]$$
- Compute the projection statistics using the Unscented method
  (11a) $$\begin{array}{cc} {\tilde{X}}_{k+1} & =\sum_{i=0}^{2n}\omega_{i}^{\mu }{\tilde{X}}_{k+1}^{\left(i\right)} \end{array}$$
  (11b) $$\begin{array}{cc} {}[{\tilde{P}}_{k+1}] & =\sum_{i=0}^{2n}\omega_{i}^{c}\left({\tilde{X}}_{k+1}^{\left(i\right)}-{\tilde{X}}_{k+1}\right){\left({\tilde{X}}_{k+1}^{\left(i\right)}-{\tilde{X}}_{k+1}\right)}^{T}+\left[Q\right] \end{array}$$

##### Measurements Projection

- Generate a weighted point set from the estimated state
  (12) $${\tilde{X}}_{k+1}=\left[{\tilde{X}}_{k+1},{\tilde{X}}_{k+1}+\sqrt{\left(n+\lambda \right)\left[{\tilde{P}}_{k+1}\right]},{\tilde{X}}_{k+1}-\sqrt{\left(n+\lambda \right)\left[{\tilde{P}}_{k+1}\right]}\right]$$
- Propagate the points through the measurement function
  (13) $${\tilde{Y}}_{k+1}^{\left(i\right)}=h\left({\tilde{X}}_{k+1}^{\left(i\right)},U_{k+1},0\right),i\in \left[0,2n\right]$$
- Estimate the mean and covariance of the measurement
  (14a) $$\begin{array}{cc} {\tilde{Y}}_{k+1} & =\sum_{i=0}^{2n}\omega_{i}^{\mu }{\tilde{Y}}_{k+1}^{\left(i\right)} \end{array}$$
  (14b) $$\begin{array}{cc} {}[{\tilde{P}}_{yy,k+1}] & =\sum_{i=0}^{2n}\omega_{i}^{c}\left({\tilde{Y}}_{k+1}^{\left(i\right)}-{\tilde{Y}}_{k+1}\right){\left({\tilde{Y}}_{k+1}^{\left(i\right)}-{\tilde{Y}}_{k+1}\right)}^{T}+\left[R\right] \end{array}$$
- Estimate the crossed covariance between the state and measurement
  (15) $$\left[{\tilde{P}}_{xy,k+1}\right]=\sum_{i=0}^{2n}\omega_{i}^{c}\left({\tilde{X}}_{k+1}^{\left(i\right)}-{\tilde{X}}_{k+1}\right){\left({\tilde{Y}}_{k+1}^{\left(i\right)}-{\tilde{Y}}_{k+1}\right)}^{T}$$

##### Correction

- Compute the Kalman Gain
  (16) $$\left[K_{k+1}\right]=\left[{\tilde{P}}_{xy,k+1}\right]{\left[{\tilde{P}}_{yy,k+1}\right]}^{-1}$$
- Correct the state
  (17a) $$\begin{array}{cc} {\hat{X}}_{k+1} & ={\tilde{X}}_{k+1}+\left[K_{k+1}\right]\left(Y_{k+1}-{\tilde{Y}}_{k+1}\right) \end{array}$$
  (17b) $$\begin{array}{cc} {}[{\hat{P}}_{k+1}] & =\left[{\tilde{P}}_{k+1}\right]-\left[K_{k+1}\right]\left[{\tilde{P}}_{yy,k+1}\right]{\left[K_{k+1}\right]}^{T} \end{array}$$

With its precise projection, the UKF is much faster to converge and gives more precise results on highly non-linear systems. This precision is possible at the expense of a far more computational estimation process.

### 2.3. Algorithm Complexity and Computing Power

As the goal of this work is to provide a real-time estimation of the physical values on embedded low-power hardware, the processing time of the used algorithms has to be taken into account during the design. In this section, the algorithmic complexity of the different kinds of Kalman filters will be discussed, and compared, taking into account the processing capabilities of common microcontrollers.

To compare the algorithm complexities, it is mandatory to choose a complexity indicator. The commonly used indicators are:

- the processed lines counts to do an operation;
- the number of Multiplication and Accumulation (MAC) operations; and
- the number of Floating point Operations (FLOP) ( i.e., the number of operation on “Real numbers” in the algorithm).

In the case of operations on microcontrollers and matrix related operations, the FLOP is the most representative indicator for the algorithms complexity. Using this indicator, the mathematical operations relative to Kalman filtering will be discussed in the next part.

#### 2.3.1. Algorithms Complexity

Kalman filtering is all about matrices and vectors operations, from the simple addition of two vectors to the inversion of a matrix. In those kinds of operations, algorithmic complexity can be expressed in relation with the vectors and or matrices dimensions.

As an example, the steps required for the computation of the average of the components of a vector of size *n* are:

- one affectation for the initialization of the sum variable;
- one addition and affectation per element (*n* addictions and affectations);
- one division; and
- one affectation for the result.

Let’s define the following complexity indicators:

- T(f(n)), the number of operations to be executed to solve the problem; and
- $O\left(f_{O}\left(n\right)\right)=\lim_{n\to +\infty }T\left(f\left(n\right)\right)$.

Then, the algorithmic complexity of the averaging operation is T(3+2n) with a complexity order of O(2n). However, as the affectations can be considered as simple operations, the results can be simplified as T(1+n), with an order of O(n).

Following the same method, we can get the complexities of every matrix operation as exposed in Table 3.

In the case of Kalman filters, the matrix inversions can be simplified by a factor of 2 as the matrix to invert is Hermitian. The Cholesky inversion can be used in this case, giving a $T(2n^{3}+n^{2})$ complexity, with an order of $O(2n^{3})$ [17].

From this point, the Kalman filters complexity are shown in Table 4, using *n* as the state vector size, *m* as the measurement vector size and *p* as the command vector size.

As a result, the Kalman filters computing complexities are summed-up in the Table 5.

Using this analysis, the UKF algorithm demands about twice the computing time of an equivalent EKF algorithm, which can be decisive in small applications. Moreover, the computational complexity of these filters grows extremely fast with the size of the system model, limiting their real-time usage to bounded complexity system model, with reasonable state, command, and measurement vectors size. It also has to be noted that the previous study does not take into account the processing time of the non-linear functions called by the EKF and UKF algorithms.

#### 2.3.2. A Computing Power Overview

Embedded systems, and thus Smart Sensors, are mainly targeting low power consumption, since most of them are battery powered, and aim for low manufacturing costs. Consequently, such systems are often designed around single core microcontroller architectures with low operating frequencies. Moreover, only high-end microcontrollers implement hardware Floating Point Unit (FPU) to accelerate the computation of “real numbers”, due to their manufacturing cost and power consumption. Recent technological advances tend to improve this part [18].

Using this knowledge, the processing power of the used controller has to be acknowledged to ensure the complexity of the filter is not too important to ensure real-time operations. As an example, the comparison can be done between three largely used microcontrollers:

- the ATMega328, a 8 bits microcontroller, the most commonly used in hobbyists designs as core controller of the Arduino Uno board;
- the STM32L053, a ultra low-consumption 32 bits microcontroller, used in the 2D orientation estimation demonstration; and
- the STM32F4xx, a high-end 32 bits microcontrollers family, embedding a FPU to accelerate computation of floating points numbers.

The computing power of those units is described in Table 6.

With this technical background, it is now possible to establish a method allowing to use Kalman filters into Smart Sensors or any other embedded system, keeping in mind the complexity problem.

## 3. The Proposed Methodology

The proposed methodology focuses on the system modelization and the algorithmic complexity containment, the main steps being discussed in this section. The next section will provide an example detailing and illustrating these steps.

### 3.1. Specify the Use-Cases

The first major step in a system design is the use-cases identification. For embedded sensors data fusion, the focus will mainly be on two parameters:

- the operation context of the system—to what end it is being used (e.g., Calibration mode, Normal estimation mode); and
- for each context, what are the parameters: known and controlled parameters, parameters to be estimated...

For the example of Calibration and Normal estimation use cases, we can sum-up the process as expressed in Table 7.

Once the use-cases have been clearly identified, the main task focusses on the system behavioral equations identification.

### 3.2. Identify the System Equations

The system modelization is mostly about behavioral equations identification. In this part, the study consists in:

- defining all the physical parameters affecting the system outputs;
- defining all the calibration parameters (i.e., the dispersion parameters due to the sensors manufacturing process), and checking if it is possible to measure them independently of the desired measurement; and
- defining all the equations linking these physical parameters (those are mainly differential equations).

### 3.3. Create the System Models for Each Use-Case

Once the use-cases and the system equations are established, the model creation part is decomposed as follows, with one model per use-case:

- the known parameters are put into the command vector $U_{k}$ of the system;
- the parameters to be estimated and all the intermediate parameters in the differential equations are put into the state vector $X_{k}$;
- the measured output values of the system are put into the measurement vector $Y_{k}$;
- the evolution and measurement equations are written according to the previously established equations; and
- the system equations time discretization for continuous time equations (as the Kalman filters only works with discrete time models).

At this point, the designer should look for uncoupled, or unidirectionally coupled subsystems into the main system, especially if this subsystem has its own measurement outputs and can be expressed as a linear subsystem. If subsystems can be identified, the designer should consider dividing the system into multiple systems, easier to process: for example, a linear system composed of two commands, seven states and five measurements will have a complexity order about O(1372), according to Equation (18a). However, if this system can be decomposed in two subsystems, one with one command, three states and two measurement, the other with two commands (one from the previous system state, for the coupling), four states and three measurements, the overall complexity drops to O(364), according to Equation (18b), which gives a 3.7 times complexity optimization.

(18a) $$\begin{array}{c} O\left(4\times 7^{3}\right)=O\left(4\times 343\right)=O\left(1372\right) \end{array}$$

(18b) $$\begin{array}{c} O\left(4\times 3^{3}\right)+O\left(4\times 4^{3}\right)=O\left(108\right)+O\left(256\right)=O\left(364\right) \end{array}$$

### 3.4. Apply an Adapted Filter

As the main goal of the design, the filter selection and implementation have to be carefully studied in order to assess the best possible performances. The designer will have to implement one Kalman filter per subsystem designed in the previous step.

To select the best possible solution for each equation, the following rules should be applied.

- If the subsystem is purely linear (i.e., its evolution and measurement equations are in the form of Equations (3) and (4)), the implemented estimator should be a Kalman filter.
- If the subsystem is purely non-linear (i.e., its evolution and measurement equation are in the form of Equations (1) and (2)), the implemented estimator should be an EKF or UKF, depending on the non-linearity, the state vector length and the available processing power.
- If the subsystem is mixed (i.e., its evolution equation is linear and its measurement equation is non-linear), the evolution part should be handled by Kalman filter implementation and the measurement part should be implemented using EKF or UKF method, in order to optimize the processing load.

Finally, to optimize the processing time, some basic equations should be rewritten to their bare minimum: for example, a linear evolution equation in the form of Equation (19a) can be simplified to a couple of operations (Equations (19b) and (19c)) ($X_{k}\left[n\right]$ being the *n*th element of the vector $X_{k}$).

(19a) $$\begin{array}{c} X_{k+1}=\left[\begin{array}{cc} 1 & 0\\ 0 & 1 \end{array}\right]\cdot X_{k}+\left[\begin{array}{cc} 0 & 1\\ 0 & 0 \end{array}\right]\cdot U_{k} \end{array}$$

(19b) $$\begin{array}{c} X_{k+1}\left[0\right]=X_{k}\left[0\right]+U_{k}\left[1\right] \end{array}$$

(19c) $$\begin{array}{c} X_{k+1}\left[1\right]=X_{k}\left[1\right] \end{array}$$

In this example, the unoptimized version is composed of:

- two matrix multiplications by a vector, of complexity T(2×2×2×1)=T(8) each; and
- an addition of two vectors of two elements, of a complexity of T(2)

The optimized version is composed of one addition of two elements and two affectations (with virtually no computational cost), which gives a complexity optimization of T(10) to T(1).

## 4. Application to a 2D Orientation Estimation Problem

The 2D orientation estimation is a common problem in robotics [19]. For instance, in a self-balancing robot, the orientation of the robot has to be accurately measured at high speed in order to control the system using a feedback loop.

### 4.1. The Sensing Elements

**Figure 4:**
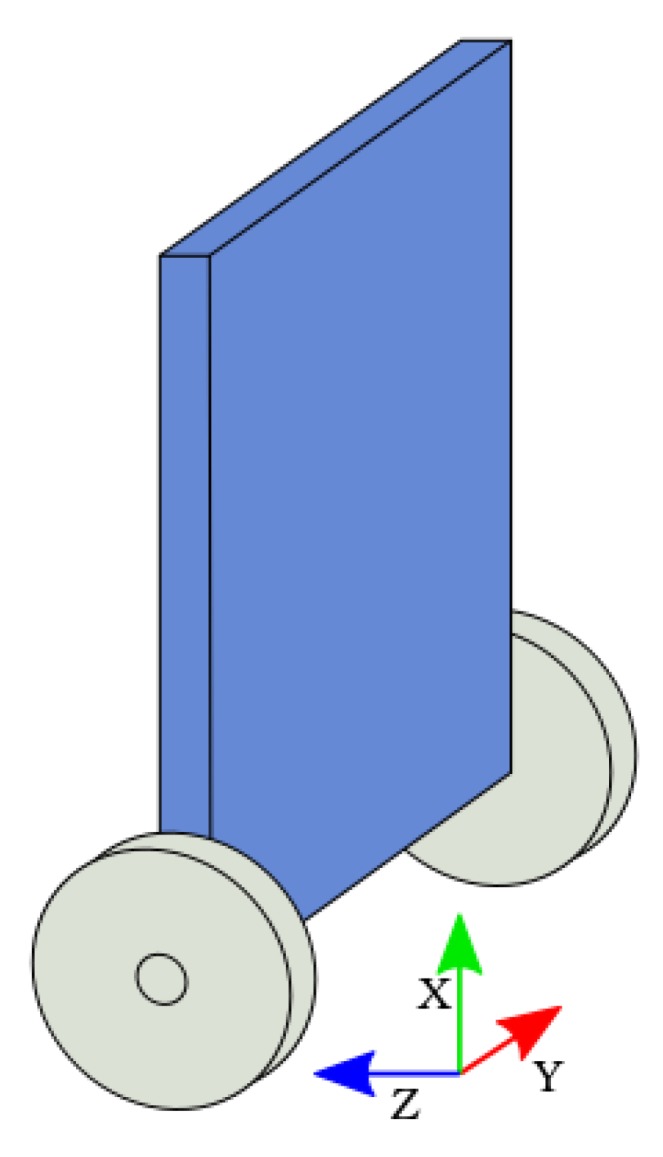
Self-balancing robot orientation frame.

To estimate the orientation of the robot in the XZ plane (Figure 4), the chosen approach relies on a three-axis accelerometer and three-axis gyroscope integrated circuit, the *LSM6DSL* sensor from *ST Microelectronics*. This circuit is used as a part of the development sensor board *X-NUCLEO-IKS01A2* [20], which can be directly plugged on the microcontroller development board.

This sensor has the following features:

- raw measurements for the Accelerations and Rotational speed on *X*, *Y* and *Z* axis;
- internal processing for free-fall detection, movement detection, 6D/4D orientation, click and double-click detection, pedometer, step detector and counter;
- an independent automatic sampling with data storage in FIFO;
- I${}^{2}$C or SPI serial interface; and
- two configurable interrupt output lines.

A corresponding driver library is also provided with the development kit for STM32 development boards.

As for all sensors, the measurements on the accelerometer and gyroscope can be biased. In the following study, only the gyroscope bias will be considered to have a significant impact on the measurement and thus will be compensated.

### 4.2. The Processing Unit

As a processing unit, the ultra-low power *STM32L053R8* has been selected, using the corresponding Nucleo development kit. This microcontroller features:

- a low-power 32 MHz, 32 bits ARM Cortex-M0 processor, without FPU;
- 64 KB of Flash and 8 KB of RAM;
- a processing power of about 180 kFLOPs/s at 32 MHz; and
- ultra low power consumption, with 88 μA/MHz running power consumption, and down to 270 nA Stand-by mode.

This microcontroller targets battery powered application, which is the main scope of the current study.

### 4.3. Applying the Methodology

The first step to design the sensor requires in the use-cases listing establishment. The proposed solution has two use-cases.

- Calibration mode: The sensor is still, on a table. Using this measurement, the gyroscope measurements should be zero, and the sensor bias is estimated by the filter.
- Orientation estimation mode: The sensor bias is known and used as a control input, and the sensor orientation is estimated by the filter.

With the use-cases established, the system equations have to be written.

#### 4.3.1. System Equations

To measure the 2D orientation of the system, the process relies on the measurement of the gravity acceleration by the accelerometers. This information is, however, sensitive to noise and parasite accelerations (e.g., chaos relative to the movement of the robot). The measurement stability is enhanced by the inclusion of gyroscope measurement, which assess the rotational speed.

**Figure 5:**
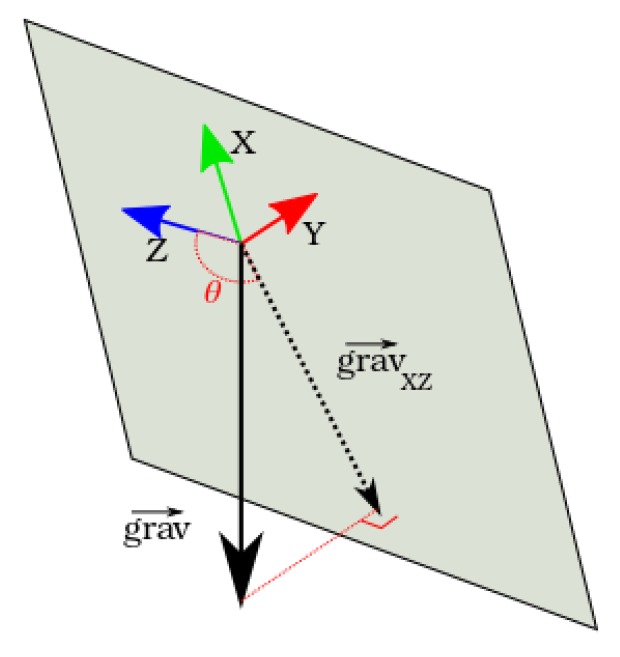
Gravity vector projection into the XZ plane.

The equations express the projection of the gravity vector in the XZ 2D plane of the robot and the measurement of its angle and norm (Figure 5).

Given this hypothesis, the three main parameters to the system are:

- the gravity vector projection norm $|grav_{XZ}|$;
- the gravity vector projection angle $\theta_{Y}$, which is the desired measurement translating the system orientation in the 2D plane; and
- the gyroscope measurement bias $b_{gyro}$.

The system noises are:

- the acceleration noise on *X* and *Z* axes: $R_{ax}$ and $R_{az}$.

From these parameters, the sensor measurements are expressed as Equation (20a) for the gyroscope and Equations (20b) and (20c) for the accelerometers.

(20a) $$\begin{array}{c} g_{Y}=\frac{\delta \theta_{Y}\left(t\right)}{\delta t}+b_{gyro} \end{array}$$

(20b) $$\begin{array}{c} a_{x}=\left|grav_{XZ}\right|*\sin\left(\theta_{Y}\right)+R_{ax} \end{array}$$

(20c) $$\begin{array}{c} a_{z}=\left|grav_{XZ}\right|*\cos\left(\theta_{Y}\right)+R_{az} \end{array}$$

The system evolution can also be expressed by Equations (21a) and (21b).

(21a) $$\begin{array}{c} \frac{\delta \theta_{Y}\left(t\right)}{\delta t}=g_{Y}-b_{gyro} \end{array}$$

(21b) $$\begin{array}{c} \frac{\delta |grav_{XZ}|\left(t\right)}{\delta t}=0 \end{array}$$

At this point, the system equations have to be established for each use-case.

##### Calibration Mode Equations

In calibration mode, the system known input is the rotational velocity: the system being still, Equation (22) is valid.

(22) $$\frac{\delta \theta_{Y}\left(t\right)}{\delta t}=0=g_{Y}-b_{gyro}$$

As the global system orientation is not relevant at this point, θ and $|g_{XZ}|$ will not be estimated in this mode, and thus $a_{x}$ and $a_{z}$ will not be monitored.

The state space equations for the calibration mode can be written as Equations (23a) and (23b).

(23a) $$\begin{array}{c} X_{cal,k+1}=\left(b_{gyro,k+1}\right)=X_{cal,k}+U_{cal,k}=\left(b_{gyro,k}\right)+0 \end{array}$$

(23b) $$\begin{array}{c} Y_{cal,k}=\left(g_{Y}\right)=X_{cal,k}=\left(b_{gyro,k}\right) \end{array}$$

When the system is stil, the gyroscope bias observation is trivial using this description.

##### Estimation Mode Equations

In estimation mode, the known input vector of the system is composed of the gyroscope bias. If the gyroscope noise is considered to be neglectable, the gyroscope *Y* axis measurement can also be added to the command vector.

Therefore, the state vector is composed of the gravity projection norm and angle. The system measurements are the accelerations along *X* and *Z* axes. The system equations can be written as Equations (24a) and (24b).

(24a) $$\begin{array}{c} {\dot{X}}_{est}\left(t\right)=\left(\begin{array}{c} \frac{\delta \theta_{Y}\left(t\right)}{\delta t}\\ \frac{\delta |grav_{XZ}|\left(t\right)}{\delta t} \end{array}\right)=\left(\begin{array}{c} g_{Y}-b_{gyro}\\ 0 \end{array}\right) \end{array}$$

(24b) $$\begin{array}{c} Y_{est}\left(t\right)=\left(\begin{array}{c} a_{X}\left(t\right)\\ a_{Z}\left(t\right) \end{array}\right)=\left(\begin{array}{c} |grav_{XZ}\left(t\right)|\cdot \sin\left(\theta \left(t\right)\right)\\ |grav_{XZ}\left(t\right)|\cdot \cos\left(\theta \left(t\right)\right) \end{array}\right) \end{array}$$

The discrete time equations can be approximated in this case to Equations (25a) and (25b) without precision loss. The sampling rate is set to 26 Hz in the current study per arbitrary choice among available hardware settings.

(25a) $$\begin{array}{c} X_{est,k+1}=\left(\begin{array}{c} \theta_{k+1}\\ |grav_{XZ,k+1}| \end{array}\right)=\left[\begin{array}{cc} 1 & 0\\ 0 & 1 \end{array}\right]\cdot \left(\begin{array}{c} \theta_{k}\\ |grav_{XZ,k}| \end{array}\right)+\left[\begin{array}{cc} \frac{1}{26} & -\frac{1}{26}\\ 0 & 0 \end{array}\right]\cdot \left(\begin{array}{c} g_{Y}\\ b_{gyro} \end{array}\right) \end{array}$$

(25b) $$\begin{array}{c} Y_{est,k}=\left(\begin{array}{c} a_{X,k}\\ a_{Z,k} \end{array}\right)=\left(\begin{array}{c} |grav_{XZ,k}|\cdot \sin\left(\theta_{k}\right)\\ |grav_{XZ,k}|\cdot \cos\left(\theta_{k}\right) \end{array}\right) \end{array}$$

Now that the equations have been established for each use-case, the Kalman filters have to be applied to those systems.

#### 4.3.2. Applying an Adapted Filter

A different filter has to be applied to each use-case, as follows.

##### Calibration Mode Filter

As this equation system is relatively simple, having a single state which translates to the single measurement, a simple low-pass filter should be used for the gyroscope bias compensation.

The selected method here was to compute the average value of the first 100 samples, and use this value as a $b_{gyro}$.

##### Estimation Mode Filter

The system equations for the Estimation mode are: linear for the evolution and non-linear for the measurement. As explained in Section 3.4, the optimal implementation uses Kalman filter equations for the evolution estimation and EKF equations for the Prediction and Correction parts (Table 8).

Where $\left[H_{X,k}\right]$ is the measurement Jacobian matrix listed in Equation (26).

(26) $$\left[H_{X,k}\right]=\left[\begin{array}{cc} \sin({\tilde{\theta }}_{k}) & |{\tilde{grav}}_{XZ,k}|\times \cos{\tilde{\theta }}_{k}\\ \cos({\tilde{\theta }}_{k}) & -|{\tilde{grav}}_{XZ,k}|\times \sin{\tilde{\theta }}_{k} \end{array}\right]$$

At this point, the covariance matrices [Q] and [R] have to be adjusted to get the best result.

As part of the optimization process, some unnecessary operations have been removed from Table 8 equations and the state evolution can be processed by only updating the needed elements. The computation of the Kalman gain can also be optimized by caching the intermediate result of $\left[{\tilde{P}}_{k}\right]{\left[H_{X,k}\right]}^{T}$, thus reducing the number of computed matrices multiplications.

## 5. Results and Discussion

The estimator has been implemented in C and deployed for the selected target. The results of every fourth estimation were sent through a virtual UART connection to the computer to be displayed in real-time.

**Figure 6:**
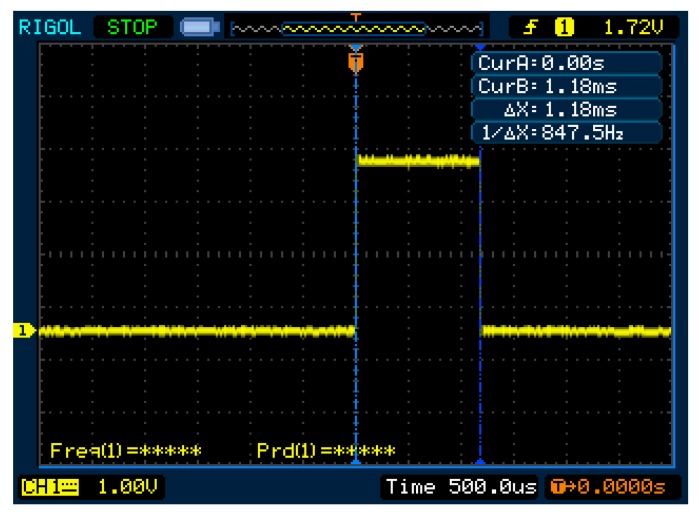
2D orientation estimation Kalman filter processing time.

There were no means available at the moment of the test to establish the precision of the measurement and assess the dynamic precision of the algorithm. It was however possible to establish the following results (Table 9):

At the microcrontroller nominal speed of 32 MHz, the CPU takes 1.18 ms to compute each estimation, executing the complete algorithm during that time slot (Figure 6). For a 26 Hz measurement rate, the CPU usage due to the estimation process is only 3.8%, giving a large amount of processing power to other tasks or to update with a more complex system model.

The large untapped processing power available came as a surprise to the writers beating best expectations, as the previous computational burden estimation for a similar project (3D pose estimation on a 9 axis IMU at a 50 Hz sampling rate, discussed in the AREM project part of [21]) were far more important with a 465 kFLOPs/s requirement using an EKF estimator without optimizations. Future studies may consider including continuous estimation of the gyroscope bias. Furthermore, the impact of the accelerometers bias and gain should be studied on the precision of the results.

Finally, the integration of the whole system model (i.e., the robot-relative behavior) should be studied and integrated into the estimation process, providing the ability for a better control loop for the global application.

## 6. Materials and Methods

All the measurement have been done on development kits available from STMicroelectronics:

- NUCLEO-L053R8 for the microcontroller development kit; and
- X-NUCLEO-IKS01A2 for sensing elements.

The code for all experiments is available on GitHub at https://github.com/wolvi-lataniere/STM32L053_performances_measurement.

Further discussions about processing power measurements and the developed 2D Orientation library are available at http://perso.imerir.com/avalade/site/index.php?view=STM32L053%202D%20Orientation%20library.

## 7. Conclusions

The primary objective of this work was to develop a systematic approach for Smart Sensors data fusion designs using Kalman filters. The main pitfall when working with such computationally expensive algorithms embedded in microcontrollers is the limited processing power available. Consequently, the proposed methodology focused on complexity aware techniques to optimize the filter equations in order to fit the low-power requirements. The described optimizations make possible bounding the complexity of the Kalman filters. It should however be noted that large system models are known to be expensive to compute and cannot be addressed at high frequencies with low-power targets.

With less than 4% of the CPU time dedicated to the filter computation, the proposed 2D orientation estimation illustration gave unexpectedly good results in terms of processing time on an ultra-low power target. As a result, the writers attend to explore the capabilities of these optimized filters on a more complex application-specific model for the self-balancing robot in the near future.

The goal for the writers is to continue applying the methodology to a wider range of Smart Sensors projects, targeting new sensing elements, lower-end microcontrollers, and more complex models [21].

## Figures and Tables

**Table 1:** Kalman filter processing steps.

Step	Kalman Filter	Real System
Evolution	${\tilde{X}}_{k+1}=\left[A\right]{\hat{X}}_{k}+\left[B\right]U_{k}$	$X_{k+1}=\left[A\right]X_{k}+\left[B\right]U_{k}+V_{k}$
	$\left[{\tilde{P}}_{k+1}\right]=\left[A\right]\left[{\hat{P}}_{k}\right]{\left[A\right]}^{T}+\left[Q\right]$	
Prediction/measurement	${\tilde{Y}}_{k}=\left[C\right]{\tilde{X}}_{k}+\left[D\right]U_{k}$	$Y_{k}=\left[C\right]X_{k}+\left[D\right]U_{k}+W_{k}$
Correction	$E_{k}=Y_{k}-{\tilde{Y}}_{k}$	
	$\left[K_{k}\right]=\left[{\tilde{P}}_{k}\right]{\left[C\right]}^{T}{\left(\left[C\right]\left[{\tilde{P}}_{k}\right]{\left[C\right]}^{T}+\left[R\right]\right)}^{-1}$	
	${\hat{X}}_{k}={\tilde{X}}_{k}+\left[K_{k}\right]E_{k}$	
	$\left[{\hat{P}}_{k}\right]=\left(I-\left[K_{k}\right]\left[C\right]\right)\left[{\tilde{P}}_{k}\right]$	

**Table 2:** EKF estimation steps.

Step	EKF	Real System
Evolution	${\tilde{X}}_{k+1}=f\left({\hat{X}}_{k},U_{k},0\right)$	$X_{k+1}=f\left(X_{k},U_{k},V_{k}\right)$
	$\left[{\tilde{P}}_{k+1}\right]=\left[F_{X,k+1}\right]\left[{\hat{P}}_{k}\right]{\left[F_{X,k+1}\right]}^{T}$	
	$+\left[F_{V,k+1}\right]\left[Q\right]{\left[F_{V,k+1}\right]}^{T}$	
Prediction/measurement	${\tilde{Y}}_{k}=h\left({\tilde{X}}_{k},U_{k},0\right)$	$Y_{k}=h\left(X_{k},U_{k},W_{k}\right)$
Correction	$E_{k}=Y_{k}-{\tilde{Y}}_{k}$	
	$\left[K_{k}\right]=\left[{\tilde{P}}_{k}\right]{\left[H_{X,k}\right]}^{T}{\left(\left[H_{X,k}\right]\left[{\tilde{P}}_{k}\right]{\left[H_{X,k}\right]}^{T}+\left[R\right]\right)}^{-1}$	
	${\hat{X}}_{k}={\tilde{X}}_{k}+\left[K_{k}\right]E_{k}$	
	$\left[{\hat{P}}_{k}\right]=\left(I-\left[K_{k}\right]\left[H_{X,k}\right]\right)\left[{\tilde{P}}_{k}\right]$	

**Table 3:** Matrix operations complexity sum-up.

Operation	T(.)	O(.)
Matrix multiplication	2×n×m×p	2×n×m×p
Adding two vectors of size *n*	*n*	*n*
Adding two matrices of size (n,m)	n×m	n×m
Transpose a matrix	0	0
Invert a matrix	$4\times n^{3}+2\times n^{2}$	$4\times n^{3}$
Mean vector of a matrix	n×(m+1)	n×m
Mean value of a vector	n+1	*n*
Covariance de deux matrices	2×n×m×p	2×n×m×p

**Table 4:** Kalman filtering complexity depending on *n*, *m* and *p*.

Algo	Opération	O(.)
(E)KF	${\tilde{X}}_{k+1}=\left[A\right]{\hat{X}}_{k}+\left[B\right]U_{k}$	$2n^{2}$
	$\left[{\tilde{P}}_{k+1}\right]=\left[A\right]\left[{\hat{P}}_{k}\right]{\left[A\right]}^{T}+\left[Q\right]$	$4n^{3}$
	${\tilde{Y}}_{k}=\left[C\right]{\tilde{X}}_{k}+\left[D\right]U_{k}$	2m(n+p)
	$E_{k}=Y_{k}-{\tilde{Y}}_{k}$	*m*
	$\left[K_{k+1}\right]=\left[{\tilde{P}}_{k}\right]{\left[C\right]}^{T}{\left(\left[C\right]\left[{\tilde{P}}_{k}\right]{\left[C\right]}^{T}+\left[R\right]\right)}^{-1}$	$4n^{2}m$/$4m^{2}n$
	${\hat{X}}_{k}={\tilde{X}}_{k}+\left[K_{k}\right]E_{k}$	2mn
	$\left[{\hat{P}}_{k}\right]=\left(I-\left[K_{k}\right]\left[C\right]\right)\left[{\tilde{P}}_{k}\right]$	∼$2n^{3}$/$2m^{2}n$
UKF	$X_{k}=\left[{\hat{X}}_{k},{\hat{X}}_{k}\pm \sqrt{\left(n+\lambda \right)\left[{\hat{P}}_{k}\right]}\right]$	$n^{3}$
	${\tilde{X}}_{k+1}^{\left(i\right)}=f\left(X_{k}^{\left(i\right)}\right)$	2nO(f(.))
	${\tilde{X}}_{k+1}=\sum_{i=0}^{2n}\omega_{i}^{\mu }{\tilde{X}}_{k+1}^{\left(i\right)}$	$4n^{2}$
	$\left[{\tilde{P}}_{k+1}\right]=\sum_{i=0}^{2n}\omega_{i}^{c}\left({\tilde{X}}_{k+1}^{\left(i\right)}-{\tilde{X}}_{k+1}\right){\left({\tilde{X}}_{k+1}^{\left(i\right)}-{\tilde{X}}_{k+1}\right)}^{T}+\left[Q\right]$	$6n^{3}$
	${\tilde{Y}}_{k+1}^{\left(i\right)}=g\left({\tilde{X}}_{k+1}^{\left(i\right)},U_{k+1},0\right)$	(2n+1)O(g(.))
	${\tilde{Y}}_{k+1}=\sum_{i=0}^{2n}\omega_{i}^{\mu }{\tilde{Y}}_{k+1}^{\left(i\right)}$	$4m^{2}n$
	$\left[{\tilde{P}}_{yy,k+1}\right]=\sum_{i=0}^{2n}\omega_{i}^{c}\left({\tilde{Y}}_{k+1}^{\left(i\right)}-{\tilde{Y}}_{k+1}\right){\left({\tilde{Y}}_{k+1}^{\left(i\right)}-{\tilde{Y}}_{k+1}\right)}^{T}+\left[R\right]$	$6m^{2}n$
	$\left[{\tilde{P}}_{xy,k+1}\right]=\sum_{i=0}^{2n}\omega_{i}^{c}\left({\tilde{X}}_{k+1}^{\left(i\right)}-{\tilde{X}}_{k+1}\right){\left({\tilde{Y}}_{k+1}^{\left(i\right)}-{\tilde{Y}}_{k+1}\right)}^{T}$	$4n^{2}m$
	${\hat{X}}_{k}={\tilde{X}}_{k}+\left[K_{k}\right]E_{k}$	2mn
	$\left[{\hat{P}}_{k}\right]=\left(I-\left[K_{k}\right]\left[C\right]\right)\left[{\tilde{P}}_{k}\right]$	∼$2n^{3}$/$2m^{2}n$

**Table 5:** Kalman filters complexity.

Algorithm	T(.)	O(.)
(E)KF	$4n^{3}+4m^{3}+6m^{2}n+4n^{2}m+3n^{2}+\cdots$	$4n^{3}$
UKF	$10n^{3}+4n^{2}m+14m^{2}n+23n^{2}+6m^{2}+\cdots$	$10n^{3}$

**Table 6:** Computing performances.

Controller	Single Precision Float Operations	Fixed Point 32 Bits Operations
ATMega328 8 bits/16 MHz	≈100,000	≈1.5 M
STM32L053 32 bits/32 MHz	≈180,000	≈3.6 M
STM32F4x 32 bits/216 MHz/FPU	≈1 M without FPU, ≈12 M	≈100 M

**Table 7:** Example of use-case specification.

Mode	Controlled/Known Parameters	Parameters to Estimate
Calibration	Main measurement parameters	System calibration parameters
Normal estimation	System calibration parameters	Main measurement parameters

**Table 8:** Filter applied to Estimation Mode.

Step	Used Equation
Evolution	${\tilde{X}}_{k+1}=\left[\begin{array}{cc} 1 & 0\\ 0 & 1 \end{array}\right]\cdot X_{k}+\left[\begin{array}{cc} \frac{1}{26} & -\frac{1}{26}\\ 0 & 0 \end{array}\right]\cdot U_{k}$
	$\left[{\tilde{P}}_{k+1}\right]=\left[\begin{array}{cc} 1 & 0\\ 0 & 1 \end{array}\right]\cdot \left[{\hat{P}}_{k}\right]\cdot \left[\begin{array}{cc} 1 & 0\\ 0 & 1 \end{array}\right]+\left[Q\right]=\left[{\hat{P}}_{k}\right]+\left[Q\right]$
Prediction/measurement	${\tilde{Y}}_{k}=\left(\begin{array}{c} {\tilde{a}}_{X,k}\\ {\tilde{a}}_{Z,k} \end{array}\right)=\left(\begin{array}{c} |{\tilde{grav}}_{XZ,k}|\cdot \sin\left({\tilde{\theta }}_{k}\right)\\ |{\tilde{grav}}_{XZ,k}|\cdot \cos\left({\tilde{\theta }}_{k}\right) \end{array}\right)$
Correction	$E_{k}=Y_{k}-{\tilde{Y}}_{k}$
	$\left[K_{k}\right]=\left[{\tilde{P}}_{k}\right]{\left[H_{X,k}\right]}^{T}{\left(\left[H_{X,k}\right]\left[{\tilde{P}}_{k}\right]{\left[H_{X,k}\right]}^{T}+\left[R\right]\right)}^{-1}$
	${\hat{X}}_{k}={\tilde{X}}_{k}+\left[K_{k}\right]E_{k}$
	$\left[{\hat{P}}_{k}\right]=\left(I-\left[K_{k}\right]\left[H_{X,k}\right]\right)\left[{\tilde{P}}_{k}\right]$

**Table 9:** Measurement performances results.

Parameter	Result
Start-up convergence time	∼30 s @ $\pm 1^{\circ }$
Still measurement noise	<0.1${}^{\circ }$
Measurement repeatability	<1${}^{\circ }$ in two consecutive tests

## Appendix A. Unscented Transform Computation

To compute the Unscented transform of a state through a non-linear function, the process steps are as follows:

- Select the 2n+1 states to propagate, those points are represented by the $X^{\left(i\right)}$ matrix, composed of 2n+1 state vectors of lenth *n*:
  (A1) $$X^{\left(i\right)}=\left\{\begin{array}{cc} \tilde{X} & ,i=0\\ \tilde{X}+\sqrt{\left(\mu +\lambda \right)\left[C_{X}\right]} & ,i\in [1,n]\\ \tilde{X}-\sqrt{\left(\mu +\lambda \right)\left[C_{X}\right]} & ,i\in [n+1,2n] \end{array}\right.$$
- Transforming the points:
  (A2) $$Y^{\left(i\right)}=f\left(X^{\left(i\right)}\right),i\in \left[0,2n\right]$$
- Define the weights:
  (A3a) $$\begin{array}{cc} \omega_{i}^{\mu } & =\left\{\begin{array}{cc} \frac{\lambda }{\lambda +n} & ,i=0\\ \frac{1}{2(\lambda +n)} & ,i\in [1,2n] \end{array}\right. \end{array}$$
  (A3b) $$\begin{array}{cc} \omega_{i}^{c} & =\left\{\begin{array}{cc} \omega_{0}^{\mu }+1-\alpha^{2}+\beta & ,i=0\\ \frac{1}{2(\lambda +n)} & ,i\in [1,2n] \end{array}\right. \end{array}$$
- Compute the mean and covariance of the result:
  (A4a) $$\begin{array}{cc} \tilde{\bar{Y}} & =\sum_{i=0}^{2n}\omega_{i}^{\mu }Y^{\left(i\right)} \end{array}$$
  (A4b) $$\begin{array}{cc} {}[{\tilde{C}}_{Y}] & =\sum_{i=0}^{2n}\omega_{i}^{c}\left(Y_{i}-\tilde{\bar{Y}}\right){\left(Y_{i}-\tilde{\bar{Y}}\right)}^{T} \end{array}$$

With parameters defined as:

- the scattering factor α around the mean value,
- the distribution relative factor β (for a Gaussian distribution β=2),
- $\lambda =\alpha^{2}\left(n+\kappa \right)-n$,
- κ the scale factor, in general the value is fixed to κ=3−n.
